# Posterior tibialis tendon transfer via the circumtibial route: a cadaveric limb analysis

**DOI:** 10.1186/s13018-014-0121-4

**Published:** 2014-11-30

**Authors:** Jian Xu, Xiang Geng, Hassan Muhammad, Xin Ma, Xu Wang, Jiazhang Huang, Chao Zhang

**Affiliations:** Department of Orthopedics, Huashan Hospital, Fudan University, No.12, Middle Wulumuqi Road, Shanghai, 200040 China

## Abstract

**Background:**

Studies have yet to determine the optimal height at which the posterior tibial tendon (PTT) can be re-routed and the tendon length discrepancy at different height levels in terms of PTT transfer via the circumtibial route. This cadaveric study was conducted to determine the optimal height of PTT subcutaneous transfer and to compare tendon length discrepancies at different heights.

**Materials and methods:**

Twenty-five fresh normal cadaveric lower legs were used for measurements. PTT was exposed and then isolated. An incision along the calf was made to re-route PTT outside the fascia. The upper edge of the incision was classified as point “a.” The distal tip of the tendon was classified as point “b.” The midpoints of the intermediate cuneiform, the lateral cuneiform, and the cuboid were defined as points “c,” “d,” and “e,” respectively. The lengths of “ab,” “ac,” “ad,” and “ae” were measured and compared at different height levels above the distal tip of the medial malleolus. Angles α, β, and γ between the tendon outside the fascia connecting to different bones and the tendon inside the fascia were also measured as tendons were transferred at different bones and different height levels. Experimental data were collected and analyzed.

**Results:**

At a height of ≥5 cm, all of the PTTs could reach the midpoints of the three bones. The lengths of ac, ad, and ae were significantly less than the length of ab (*p* < 0.05). At a height of 10 cm, angles α, β, and γ were 177° ± 2.1°, 170° ± 3.1°, and 164° ± 3.7°, respectively. These angles were not significantly different from those at a height of 11 cm (*p* >0.05).

**Conclusions:**

PTT transfer via the subcutaneous route could achieve an adequate length to be transferred to the intermediate cuneiform, the lateral cuneiform, and the cuboid from a height of 5 cm above the distal tip of the medial malleolus. A height of 10 cm could be optimal for PTT transfer in the three bones via the subcutaneous route.

## Introduction

The posterior tibial tendon (PTT) can be subjected to anterior transfer, a surgical procedure performed to repair foot drop caused by several conditions, such as irreversible lesions in the peroneal nerve or the dorsiflexor muscles of the foot and the ankle [1-5], supinated equinovarus foot deformity secondary to club deformity [6], Charcot-Marie-Tooth disease [7], leprosy [7,8], mononeuropathy, trauma to the common peroneal nerve [8-10], cerebrovascular accident [11], and Duchenne’s muscular dystrophy [12]. This procedure was first described by Ober in 1933 to treat foot drop via a circumferential route [7]. In 1954, the same transfer procedure was accomplished via an interosseous route [13-15]. These two transfer modes aim to restore dorsiflexion of the foot and normal heel-toe gait [16]. However, the surgical procedure (circumtibial (CT) versus interosseous (IO)) that provides the optimal clinical results remains the subject of debates [17].

The pathway along an interosseous membrane possibly provides a physiological direction at which a tendon is pulled, increases dorsiflexion strength, and decreases the probability of varus recurrence [12,18-20]. Although the interosseous route provides more advantages [7,13,20,21], the circumtibial route can provide beneficial effects and cover a wide range of applications. This route is safe and convenient [4]; furthermore, this route possibly contains a longer moment arm that increases mechanical advantage with reference to power [21,22] than other routes. The circumtibial route can also be reserved for patients with a calcified and unyielding interosseous membrane and those who usually experience recurrent foot inflammation and infection [19].

Studies have shown tendon length discrepancy and optimal height for transfer via an interosseous route [23]. However, studies have yet to determine whether the circumtibial transfer of the PTT to the dorsum of the foot can result in an adequate tendon length and the optimal height necessary to re-route. Some study has demonstrated that tendon bifurcation should be at least 3 cm above the ankle such that the line of pull is as close to the vertical line as possible [19]. However, studies have poorly described the details by which transfer is performed. Thus, a cadaveric study was performed to determine the optimal height level for tendon transfer and compare different height levels.

## Materials and methods

For inclusion of cadavers in the study, the written informed consents were obtained from family members or legal guardians. In addition, all human studies were approved by the China Ethics Committee and performed in accordance with the ethical standards. Twenty-five freshly frozen cadaveric lower legs which were free of previous injuries or pathology were disarticulated at the level of the knee joints and used in this study. The legs were obtained from adult cadaveric specimens, in which 14 were male (aged 34 years to 69 years with an average age of 59.6 ± 6.3 years) and 11 were female (aged 30 years to 72 years with an average of 63.7 ± 5.1 years). No evident difference was observed among males and females in terms of age (*p* > 0.05). All of the proximal origins of the posterior tibialis muscle of the limbs were left intact.

**Figure 1:**
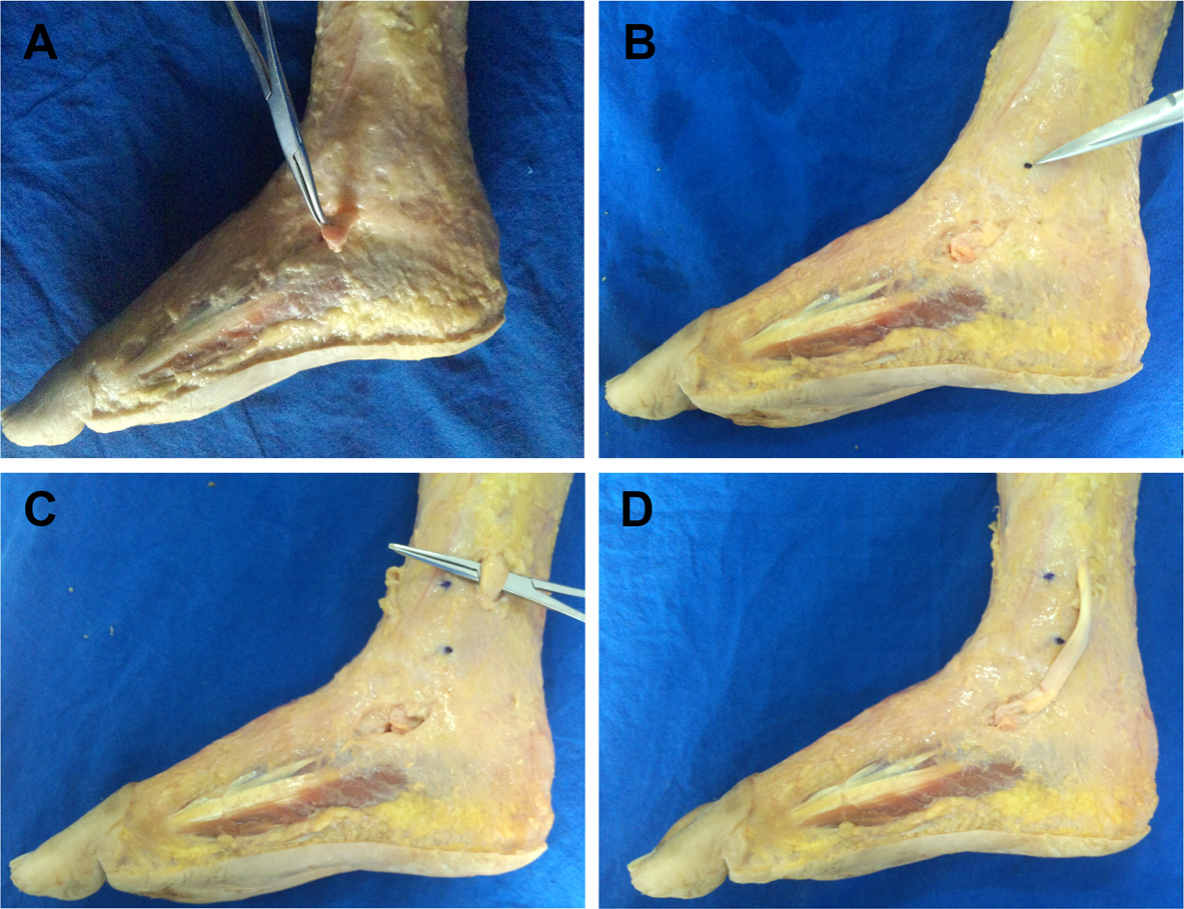
**Procedures used to expose and isolate the PTT. (A)** With a 2-cm medial incision, the tendon was divided at its insertion to the navicular region, preserving as much length as possible. **(B)** The distal tip of the medial malleolus was confirmed with a small screw as a marker. **(C)** A second 2-cm long incision posterior to the medial region of the tibial crest was made along the fascia of the calf, where the upper edge was 3 cm proximal to the distal tip of the medial malleolus. **(D)** PTT was isolated and then re-routed.

**Figure 2:**
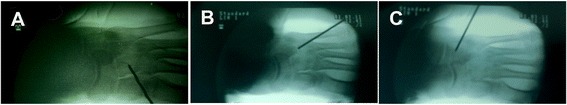
**An X-ray machine was used to determine the midpoints of the (A) intermediate cuneiform, (B) lateral cuneiform, and (C) cuboid.**

**Figure 3:**
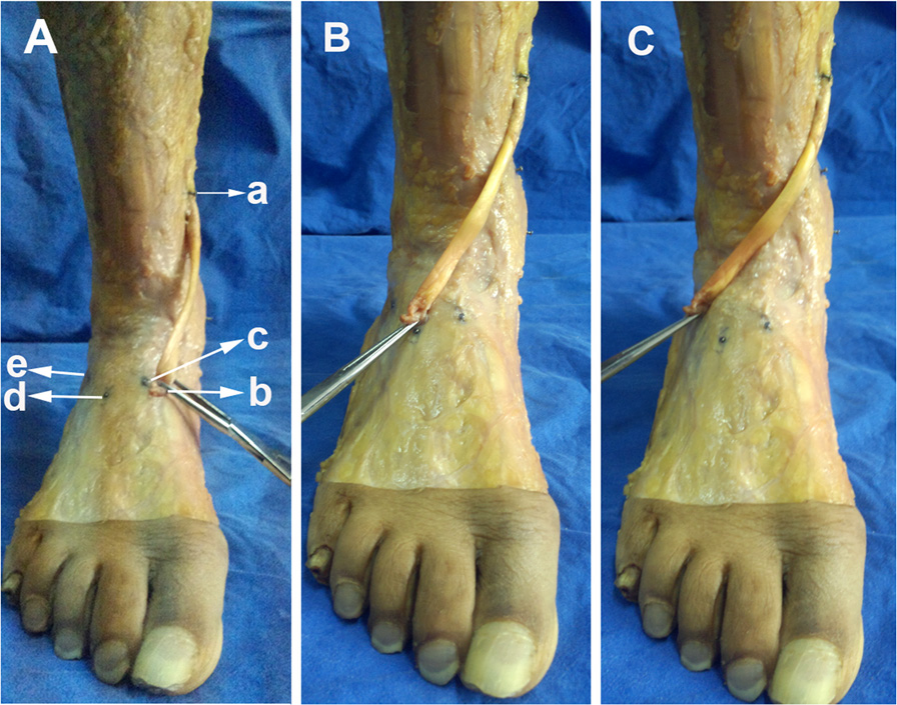
**Procedures used for measurement. (A)** We classified the point of the upper edge of the second incision as point “a,” which can be changed according to different height levels for transfer. The distal tip of the tendon was classified as point “b.” The midpoints of the intermediate cuneiform, the lateral cuneiform, and the cuboid were defined as points “c,” “d,” and “e,” respectively. PTT was re-routed to reach the midpoints of the **(A)** intermediate cuneiform, **(B)** the lateral cuneiform, and **(C)** the cuboid by applying tension to some extent. During this procedure, the limb was kept in a neutral position.

The legs were placed in neutral positions by implanting a steinmann pin from the calcaneous through the talus to the tibia. The angle of ankle dorsiflexion is kept at 0° in the horizontal plane. Then the skin was removed meticulously to simulate subcutaneous PTT transfer. At a 2-cm medial incision on the skin of navicular tuberosity, the tendon was divided at its insertion to the navicular region, but the length was maintained as much as possible, although osseous materials were retained (Figure 1A). The distal tip of the medial malleolus was confirmed using a small screw as a marker (Figure 1B). A second incision with a length of 2 cm and posterior to the medial aspect of the tibial crest was made along the medial fascia of the calf, in which the upper edge was 3 cm proximal to the distal tip of the medial malleolus (Figure 1C). Underneath the medial fascia, the PTT was isolated and retrieved; no tendinosis was found in all of the specimens. The PTT was white, hard, and tough; no adhesion was observed. The PTT was re-routed (Figure 1D). The X-ray machine was then used to determine the midpoints of the intermediate cuneiform, the lateral cuneiform, and the cuboid (Figure 2A, B, C), which were also marked using an X-ray. In this study, the point of the upper edge of the second incision was classified as point “a” to describe the length for comparison. This point can be changed according to different height levels for transfer. The distal end of the tendon was classified as point “b.” The midpoints of the intermediate cuneiform, the lateral cuneiform, and the cuboid were defined as points “c,” “d,” and “e,” respectively (Figure 3A). PTT was then re-routed to reach the midpoints of the intermediate cuneiform, the lateral cuneiform, and the cuboid by applying 10 N pre-tension; during this procedure, the limb was placed in a neutral position (Figure 3B, C). We fixed one end of a suture to the point of “a,” then pulled this suture to the points of “b,” “c,” “d” and made makers on the suture, respectively. The lengths of “ab,” “ac,” “ad,” “ae” were measured by determining the length of part of the suture which began from one end to the marker points, respectively. All the lengths of “ab,” “ac,” “ad,” and “ae” were individually measured. If the length of PTT was sufficient to reach the midpoints of the three bones, an angle was measured between the tendon outside the fascia connecting to the different bones and inner layers. To prevent the muscle-tendon force distribution, we placed the line of pull as close to the vertical line as possible. To measure the angle produced by tendon bifurcation at the tibial surface, we put a suture in the middle surface along the tendon. Then the angles produced by the suture bifurcation were measured by a protractor. The angle scan was used to determine the extent of the distributed force. Angles were denoted as α, β, and γ for the intermediate cuneiform, the lateral cuneiform, and the cuboid, respectively. After the angles at 3 cm to the distal tip of the medial malleolus were measured, the incision was extended upward in incrementing intervals of 1 cm.

Lengths “ab,” “ac,” “ad,” and “ae” and angles α, β, and γ changed according to different height levels of the proximal edge of the second incision. All of the data were measured and collected from 3 cm to 11 cm from the proximal tip to the distal tip of the medial malleolus. The experiment was finished within 2 weeks after data were collected. This procedure was also completed as soon as possible for each defrosted cadaver, to minimize the exposure of cadavers and prevent them from denaturation that may affect the accuracy of measurement.

### Statistical analysis

Statistical analysis was performed using SPSS 20.0. The results were presented as the mean with standard deviation (SD). A significant difference was defined as *p* < 0.05. Comparisons among lengths of “ab,” “ac,” “ad,” and “ae” and angles of “α, β, and γ” were analyzed by one-way ANOVA.

## Results

Among the 25 cadavers, the PTTs were pulled to the midpoints of the intermediate cuneiform, the lateral cuneiform, and the cuboid via the subcutaneous pathway in all specimens. When obtaining the PTT tendon at a height of 3 cm proximal to the tip of the medial malleolus, no PTT tendon could reach any of the insertion points (Table 1). While at a height of 4 cm, PTTs could reach the midpoints of the middle cuneiform; the difference between “ab” and “ac” lengths was not significant (*p* > 0.05; Table 1). And all of the PTTs could not reach the midpoints of the lateral cuneiform and the cuboid at this height; “ab” length was significantly less than “ac” and “ae” lengths (*p* < 0.05; Table 1). At a height of ≥5 cm, all of the PTTs could reach the midpoints of the three bones; “ac,” “ad,” and “ae” lengths were significantly less than “ab” length (*p* < 0.05; Table 1). Not all the PTT muscle and the tendons of cadaveric specimens could provide the same length for the transfer because of individual differences; however, generality was determined. During the experiments, the PTT muscle in many specimens was slightly torn and may affect muscle strength when PTT was re-routed at a height of 10 cm proximal to the distal tip of the medial malleolus. At a height of 11 cm, the PTT muscle of most cadavers was torn; as height increased, the risk of PTT tearing increased. As such, the measurements of the upper levels were terminated.

**Table 1:** **Comparison of the length of PTT tendon and the length needed for transfer**

**Height of level (cm)**	**Length (cm)**				***D***_**ab−ac**_	***p***	***D***_**ab−ad**_	***p***	***D***_**ab−ae**_	***p***
	**ab**	**ac**	**ad**	**ae**						
3	9.2 ± 2.1	9.8 ± 1.7	10.1 ± 2.3	10.8 ± 1.4	0.6 ± 2.4	0.022*	0.9 ± 2.1	0.013*	1.6 ± 1.7	0.005*
4	10.2 ± 2.1	10.3 ± 2.0	10.5 ± 1.9	11.4 ± 2.2	0.1 ± 0.2	0.13	0.3 ± 1.1	0.021*	1.2 ± 1.8	0.0089*
5	11.2 ± 2.1	10.1 ± 2.3	10.7 ± 2.5	11.1 ± 1.7	−1.1 ± 1.0	0.017*	−0.5 ± 1.7	0.020*	−0.1 ± 0.8	0.026*
6	12.2 ± 2.1	10.4 ± 2.8	11.5 ± 1.6	11.9 ± 2.0	−1.8 ± 1.4	0.009*	−0.7 ± 2.4	0.015*	−0.3 ± 1.3	0.010*
7	13.2 ± 2.1	10.9 ± 1.9	12.1 ± 1.4	12.8 ± 2.3	−2.3 ± 1.8	0.007*	−1.1 ± 1.3	0.023*	−0.4 ± 1.7	0.019*
8	14.2 ± 2.1	12.8 ± 1.3	13.1 ± 1.9	13.5 ± 2.1	−1.4 ± 2.2	0.019*	−1.1 ± 1.9	0.009*	−0.7 ± 0.6	0.031*
9	15.2 ± 2.1	13.1 ± 2.0	13.5 ± 1.6	14.0 ± 2.5	−2.1 ± 1.7	0.012*	−1.7 ± 2.1	0.014*	−1.2 ± 2.0	0.024*
10	16.2 ± 2.1	13.5 ± 2.1	14.3 ± 2.6	14.7 ± 1.1	−2.7 ± 1.9	0.016*	−1.9 ± 2.3	0.012*	−1.5 ± 1.8	0.019*
11	17.2 ± 2.1	14.1 ± 2.3	14.9 ± 1.8	15.2 ± 1.3	−3.1 ± 1.4	0.005*	−2.3 ± 2.6	0.006*	−2.0 ± 2.3	0.008*

*Significance (*p* < 0.05), ab compared with ac, ad, and ae. *D*_ab-ac_, *D*_ab-ad_, and *D*_ab-ae_ stand for the length of ab minus ac, ad, and ae, respectively.

PTT should be as straight as possible from the origin to the insertion to avoid scattering tendon force. However, this tendon cannot be straightened along the subcutaneous route for PTT transfer. As such, the angle produced by tendon bifurcation at the tibial surface should be as large as possible to straighten PTT. α, β, and γ angles were measured from 5 to 11 cm of the height proximal to the distal tip of the medial malleolus. The results are listed in Table 2. At a height of 10 cm, α, β, and γ angles were 177° ± 2.1°, 170° ± 3.1°, and 164° ± 3.7°; respectively, these angles were not significantly different from those at a height of 11 cm (*p* >0.05; Table 2).

**Table 2:** **Angles between the tendon outside the fascia to different bones and inside the fascia to different bones at different heights while transferring**

**Height of level (cm)**	**Angle (°)**		
	**α**	**β**	**γ**
3	-	-	-
4	-	-	-
5	130 ± 5.6	125 ± 7.1	120 ± 4.8
6	145 ± 6.1	137 ± 8.3	133 ± 7.1
7	152 ± 4.5	145 ± 4.8	140 ± 3.9
8	161 ± 6.8	155 ± 7.6	145 ± 5.7
9	168 ± 4.2	160 ± 5.1	154 ± 4.9
10	177 ± 2.1	170 ± 3.1	164 ± 3.7
11	178 ± 1.8*	171 ± 2.4*	165 ± 3.3*

*Significance (*p* > 0.05), “α,” “β,” and “γ” at a height of 11 cm compared with “α,” “β,” and “γ” at a height of 10 cm.

## Discussion

PTT transfer has been applied to treat various disorders related to weak ankle dorsiflexion and restore normal heel-toe gait. After PTT is transferred to the anterior region of the ankle, dorsiflexion is restored and the deforming force on the medial region of the foot is removed [1,23]. Although an interosseous route is recommended to achieve a direct pull, high dorsiflexory strength, and low probability of varus recurrence than other routes [2,8,19,20,24], the subcutaneous route is preferred for patients with a calcified and unyielding interosseous membrane; these patients usually include the elderly with recurrent foot inflammation and infection [19,25,26]. Other common problems include risks of vascular injuries and adhesions in the interosseous membrane [27]. The subcutaneous route is technically easier and less risky than the interosseous route; furthermore, this technique contains a longer moveable arm to increase mechanical advantage with respect to power, but movement range decreases [21]. However, the tendon may scar down easily in the interosseous tunnel, thereby decreasing power and effectiveness. A few studies have compared the clinical results of these two methods. Hill [28] presented that 25° to 30° can be achieved in the subcutaneous route, whereas only 17° can be achieved in the interosseous route. Bisla et al. [18] reported a 70% success rate in the interosseous route and only 29% in the subcutaneous route [28], which did not involve the relocation of the insertion site. Clinical results reported by Sores [19] and Partheebarajan [5] showed that interosseous route is better than the subcutaneous route when foot drop is corrected in leprosy cases. Das [29] reported that the pre-selection of transfer route, whether CT or IO, based on peroni strength avoids the complication of iatrogenic inversion. However, studies have reported the outcome of a single route; limited data are available regarding the two routes, particularly when these processes are conducted by the same team, in the same period. No convincing guidelines have been presented regarding the time at which a route is selected. Hence, the most efficient route remains a subject of debate.

Studies have not yet revealed the optimal site of insertion. For instance, the PTT insertion sites in the dorsum of the foot may vary according to the unequal force between the medial and lateral sides, but the intermediate cuneiform is considered as an optimal site [30,31]. Goh et al. [17] used a cadaver model to verify that insertion in the lateral cuneiform can provide optimal dorsiflexion, in which the least supination and pronation are detected. Therefore, transfers should be conducted in the midline of the foot either in the intermediate cuneiform or in the lateral cuneiform. Other studies [3,4] have focused on PTT transfer to other tendons, such as extensor halluces longus, extensor digitorum, and peroneus tertius tendons. Compared with tendon-to-bone (TtB) technique, tendon-to-tendon (TtT) suturing has been described as an alternative to the TtB procedure to eliminate the need for screws, staples, or pullout wires tied over a button [18], particularly when an insufficient PTT length is measured for the TtB procedure. However, TtT suture is surgically demanding; the complexity of reconstruction and the angle between PTT and recipient tendons dissipate the pull strength of the PTT, thereby reducing dorsiflexion power [32]. Ryssman [23] noted that a transferred tendon should be secured with a TtB interface whenever possible, and this procedure is more physiologically effective than other methods. The biggest disadvantage of TtB procedure is inadequate length as described in previous studies [17,33,34]. However, this drawback can be prevented according to our study. In the present study, the midpoints of the intermediate cuneiform, the lateral cuneiform, and the cuboid were used as the transferred sites of insertion. These sites may be potentially applied in clinical surgeries.

Studies have not yet described the optimal height at which a tendon should be re-routed, the specific length of the tendon used for insertion, and the optimal height in subcutaneous transfer. Our study indicated that tendon length when obtained at a height of 5 cm above the distal tip of the medial malleolus was sufficient for transfer. Hence, a high tendon length could be relatively efficient for transfer. However, a tendon removed from the medial fascia poses a high risk of torn PTT muscle. In our study, the PTT muscle began tearing at a height of 10 cm above the distal tip of the medial malleolus. However, the height is not as high as expected; because the optimal height for PTT re-routing is 15 cm proximal to the distal tip of the medial malleolus. After we pulled PTT muscle out at a height of 10 cm proximal to the distal tip of the medial malleolus and applied a pre-tension to PTT, PTT muscle began tearing. Tearing occurred because an extra distance is present, and this distance should be bypassed on the surface of the tibia compared with the interosseous route. We also measured the angle between the tendons outside and inside the fascia. α, β, and γ angles (the angles made by PTT with different bones) at a height of 10 cm did not significantly differ from those at a height of 11 cm (*p* > 0.05). Therefore, the height at 10 cm above the distal tip of the medial malleolus could be optimal for subcutaneous PTT transfer. However, the located sites of PTT insertion into the dorsum of the foot and the optimal height for transfer can vary according to the extent of deformity, patients’ local calf conditions, or practical requirements according to different surgeons.

All of the experimental studies using cadaveric specimens exhibit limitations. Although cadavers are freshly frozen, the devitalized muscle may be longer than that of a living body because of complete relaxation. Our study only included 25 specimens, resulting in deviations in the statistical results because of a relatively small sample size.

## Conclusions

PTT transfer via the subcutaneous route could achieve an adequate length to be transferred to the intermediate cuneiform, the lateral cuneiform, and the cuboid from a height of 5 cm above distal tip of the medial malleolus. A height of 10 cm could be optimal for PTT transfer in the three bones via the subcutaneous route.
